# Immunotherapy biomarkers in brain metastases: insights into tumor microenvironment dynamics

**DOI:** 10.3389/fimmu.2025.1600261

**Published:** 2025-08-13

**Authors:** Mu Li, Yi Zhang, Duo Yu, Yaoyu Yu, Wenxue Ma

**Affiliations:** ^1^ Department of Neurosurgery, Dengzhou People’s Hospital, Dengzhou, Henan, China; ^2^ Department of Biopharmaceutics, School of Pharmacy, Air Force Medical University, Xi’an, China; ^3^ Department of Neurosurgery, Brain Hospital Affiliated to Tongji University, Shanghai, China; ^4^ Department of Medicine, Sanford Stem Cell Institute, and Moores Cancer Center, University of California San Diego, La Jolla, CA, United States

**Keywords:** biomarkers, immunotherapy, brain metastases, cytokines, tumor microenvironment (TME), transcriptomics

## Abstract

Brain metastases (BM), represent the most common intracranial malignancies in adults and remain a major clinical challenge due to their poor prognosis and limited therapeutic options. Although immunotherapy has emerged and offers a promising strategy, its efficacy in BM is often compromised by the immunosuppressive tumor microenvironment (TME) and limited immune cell infiltration. This review highlights the critical role of cytokines and growth factors as key modulators of immune dynamics in the TME, exploring their utility as predictive and prognostic biomarkers for immune response. We examine selected categories of biomarkers including genomic, proteomic, immunological, circulating, and microenvironmental, specifically through the lens of cytokine and growth factor regulation. Additionally, we highlight how advanced technologies, including next-generation sequencing (NGS), single-cell RNA sequencing (scRNA-seq), spatial transcriptomics, and liquid biopsies, contribute to the identification and validation of these biomarkers. By addressing current challenges and proposing future directions, this review underscores the translational value of cytokine- and growth factor-related biomarkers in optimizing precision immunotherapy for patient s with BM.

## 1 Introduction

Brain metastases (BM) are among the most devastating complications in cancer patients, with lung cancer, breast cancer, and melanoma accounting for the majority of cases (1, 2). These secondary brain tumors significantly compromise neurological function and overall survival, representing a critical unmet need in oncology (3, 4). Although advances in imaging and systemic therapies have prolonged survival in patient with advanced malignancies, they have also contributed to a rising incidence of BM, emphasizing the urgency for more effective therapeutic strategies to improve patient outcomes (3, 5).

Historically, patients with BM were excluded from early immunotherapy clinical trials due to concerns about blood-brain barrier permeability and potential neurotoxicity. As a result, data on immunotherapy efficacy in this population have remained limited and often inconclusive (6, 7).

Immunotherapy has emerged as a transformative approach, offering renewed hope for patients with BM (8, 9). However, its clinical success remains hindered by the heterogeneity of BM and the highly immunosuppressive tumor microenvironment (TME). Within this context, cytokines and growth factors play pivotal roles, modulating immune cell infiltration, tumor progression, and therapeutic resistance (10, 11). Key mediators, such as interleukins, interferons, and chemokines, orchestrate immune responses, while factors such as vascular endothelial growth factor (VEGF) promotes angiogenesis and support an immunosuppressive niche (11, 12).

Biomarkers derived from cytokine and growth factor pathways offer substantial potential for guiding and refining immunotherapy strategies. Genomic alterations, proteomic profiles, and immunological markers such as PD-L1 expression and tumor-infiltrating lymphocytes (TILs), provide valuable insights into the molecular and immune landscape of BM (13, 14). Emerging platforms like next-generation sequencing (NGS), single-cell RNA sequencing (scRNA-seq), and liquid biopsy platforms, has further refined the discovery and validation of these biomarkers, facilitating their translation into clinical applications (15–17).

This review explores the evolving landscape of immunotherapy biomarkers in BM, focusing specifically on cytokines and growth factors and their regulatory roles with in the TME. By addressing existing knowledge gaps and outlining future directions, we aim to highlight the translational potential of biomarker-guided strategies in advancing precision immunotherapy for patients with BM.

## 2 Current landscape of biomarkers in BM

Biomarkers are critical for understanding and treating BM by providing insights into their genetic, proteomic, immunological, and microenvironmental characteristics. This section explores key categories of biomarkers relevant to BM, including genomic, proteomic, immunological, circulating, and microenvironmental markers.

### 2.1 Genomic biomarkers

Genomic biomarkers illuminate the molecular drivers of BM and enable the development of targeted therapies. Common alterations, including mutations in epidermal growth factor receptor (EGFR), anaplastic lymphoma kinase (ALK), and B-Raf proto-oncogene serine/threonine kinase (BRAF), are frequently observed in metastases derived from primary lung, breast, and melanoma tumors (18, 19). These mutations influence tumor progression, prognosis, and responsiveness to therapies. Additionally, genes encoding cytokines and growth factor receptors, such as vascular endothelial growth factor (VEGF) and interleukin-6 (IL-6), have been implicated in promoting angiogenesis and contributing to immune evasion within the TME (20, 21). Next-generation sequencing (NGS) enables comprehensive profiling these genetic alterations, guiding therapeutic decisions and refining precision oncology approaches (22).

### 2.2 Proteomic biomarkers

Proteomic biomarkers reveal protein expression patterns associated with BM, offering insights into tumor behavior and therapy resistance. Proteins such as matrix metalloproteinases (MMPs) and VEGF are linked to angiogenesis and tumor invasion, and aggressive disease phenotypes (23, 24). Advances in mass spectrometry (MS) have facilitated the identification of differentially expressed proteins that may serve as therapeutic targets (25). Additionally, cytokine-induced proteins, including intercellular adhesion molecule-1 (ICAM-1) are emerging as indicators of tumor progression and immune modulation, although not all are directly linked to EGFR pathways (26). Integrating proteomic data with genomic and immunological markers enhances the multidimensional understanding of tumor biology (27, 28).

### 2.3 Immunological biomarkers

The immune landscape of BM is shaped by a complex network of cytokines and growth factors that orchestrate anti-tumor responses and immune evasion mechanisms (29). Key immunological biomarkers such as programmed cell death ligand 1 (PD-L1) and tumor-infiltrating lymphocytes (TILs) are widely used to predict responsiveness to immune checkpoint inhibitors (ICIs) (30, 31). Cytokines like interferon-gamma (IFN-γ) play a regulatory role in PD-L1 expression and correlate with better response to ICIs (32, 33). Additionally, the quantity and composition of TILs, particularly cytotoxic T cells (CTLs) and regulatory T cells (Tregs), are influenced by cytokines such as IL-10 and transforming growth factor beta (TGF-β), both of which contribute to immune suppression and tumor tolerance within the TME (34, 35). Recent advances in technologies single-cell RNA sequencing (scRNA-seq) and high-dimensional immunophenotyping have enhanced the ability to delineate immune cell heterogeneity and cytokine-driven subpopulations in BM, offering novel insights into immunophenotyping biomarkers (36, 37).

### 2.4 Circulating biomarkers

Circulating biomarkers provide a non-invasive means to monitor BM progression and treatment response (38, 39). Key biomarkers include circulating tumor DNA (ctDNA), exosomes (Exs), and soluble cytokines. ctDNA reflects tumor-specific mutations and copy number alterations, while exosomes (Exs) carry tumor-derived proteins and RNA (40, 41). Circulating cytokines, including IL-6, IL-8, and tumor necrosis factor-alpha (TNF-α), correlate with systemic inflammation, tumor burden, and immunotherapy resistance (42, 43). Advances in liquid biopsy technologies continue to improve sensitivity and specificity in detecting these markers (41, 44).

### 2.5 Microenvironmental biomarkers

The TME in BM is a dynamic ecosystem that mediates immune escape, therapy resistance, and tumor progression (29, 45). Key microenvironmental biomarkers include cytokines, chemokines, and stromal factors that reflect and shape immune dynamics. Elevated levels of IL-6 and TNF-α promote a pro-tumorigenic inflammatory state, while VEGF drives angiogenesis and hampers immune infiltration (46, 47). VEGF facilitates angiogenesis and disrupts immune surveillance, immunosuppressive mediators like TGF-β contributes to immunosuppressive niches (23, 48). Chemokines such as C-X-C motif chemokine ligand 12 (CXCL12) regulate spatial immune cell recruitment and promote tumor-immune crosstalk within the brain TME (42, 49). Targeting these microenvironmental signals, particularly those involving cytokine and chemokine axes, may enhance the efficacy of immunotherapies by reversing immune suppression and enabling T cell infiltration (42, 50).

The diverse array of biomarkers in BM underscores the complexity of tumor-immune interactions and highlights the importance of multi-modal strategies for personalized immunotherapy. Future research should prioritize validating these biomarkers across diverse cohorts and integrating them into clinical practice. Future efforts should focus on validating these biomarkers in diverse patient cohorts and integrating them into clinical workflows to enhance diagnostic accuracy, treatment selection, and patient outcomes (12, 51).

**Table 1** provides a consolidated overview of the major biomarker categories discussed above, highlighting representative examples, their clinical relevance, and associated references in the context of BM.

**Table 1:** Categories of biomarkers in BM.

Category	Examples	Relevance	Tumor types	References
Genomic	EGFR, ALK, BRAF mutations, VEGF, IL-6	Guides therapeutic targeting and informs tumor progression and immune evasion pathways.	NSCLC, melanoma, breast	(18–21)
Proteomic	VEGF, MMPs, ICAM-1	Indicates aggressiveness, angiogenesis, and therapeutic resistance.	NSCLC, breast, melanoma	(23–26)
Immunological	PD-L1, TILs, IFN-γ, IL-10, TGF-β	Predicts immune checkpoint inhibitor (ICI) efficacy and immune responses within the TME.	NSCLC, melanoma	(14, 34, 35, 42)
Circulating	ctDNA, Exs, IL-6, TNF-α	Enables non-invasive monitoring of disease progression and therapeutic resistance.	NSCLC, breast, melanoma	(41–44)
Microenvironmental	IL-6, TNF-α, VEGF, CXCL12	Highlights immune suppression, angiogenesis, and immune cell recruitment within the TME.	NSCLC, breast, melanoma	(23, 46–48)

## 3 Emerging biomarkers for immunotherapy

The rapid advancement of molecular and cellular technologies has significantly enhanced biomarker discovery for immunotherapy in BM (13, 52, 53). These emerging biomarkers provide opportunities for personalized medicine, enabling improved prediction of treatment responses and therapy customization (54–56). This section focuses on advanced approaches that are shaping biomarker discovery, particularly those related to cytokines and growth factors.

### 3.1 Integration of NGS and scRNA-seq

NGS and scRNA-seq are complementary technologies that together provide a comprehensive view of the tumor and immune landscape. NGS enables high-throughput identification of genetic alterations, such as mutations in VEGF, IL-6, and TGF-β, which play pivotal roles in the TME and influence immunotherapy responses (57, 58). By offering detailed genomic profiles, NGS facilitates the identification of actionable targets and enhances patient stratification for tailored treatments (59, 60).

ScRNA-seq adds another layer of granularity by analyzing gene expression at the single-cell level, uncovering rare cell populations and their functional states (36, 61). For instance, tumor-associated macrophages (TAMs) producing IL-10 or TGF-β contribute to immunosuppression, while CD8^+^ T cells secreting IFN-γ exhibit anti-tumor activity (62, 63). Together, these tools enable a multifaceted view of tumor heterogeneity and cytokine-driven dynamics, paving the way for developing precise and adaptive immunotherapy strategies (64).

**Figure 1:**
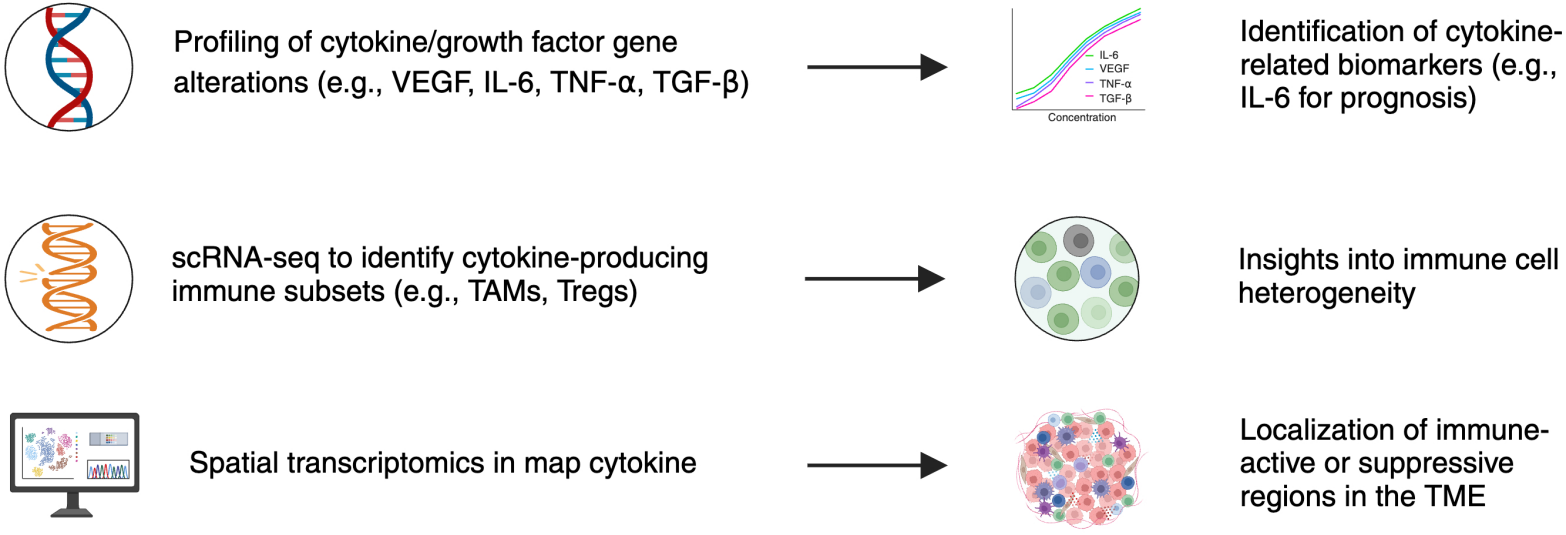
Integration of multi-omic technologies in cytokine- and growth factor-driven biomarker discovery for brain metastases. This schematic illustrates how next-generation sequencing (NGS), single-cell RNA sequencing (scRNA-seq), and spatial transcriptomics each contribute unique insights into tumor and immune biology. NGS enables profiling of gene alterations in cytokine and growth factor pathways (e.g., VEGF, IL-6, TNF-α, TGF-β), supporting biomarker identification such as IL-6 for prognosis. ScRNA-seq identifies cytokine-producing immune subsets (e.g., TAMs, Tregs), revealing immune cell heterogeneity. Spatial transcriptomics localizes cytokine activity within the TME, distinguishing immune-active or immunosuppressive regions. Together, these platforms enable mechanistic insight and translational application in precision immunotherapy.

To better understand the complexity of cytokine- and growth factor–mediated signaling in BM, **Figure 1** illustrates how integrated technologies such as NGS, scRNA-seq, and spatial transcriptomics converge to uncover relevant biomarkers, enabling both mechanistic insight and translational application in immunotherapy. **Figure 1** illustrates the integration of NGS, scRNA-seq, and spatial transcriptomics, showcasing their complementary roles in uncovering tumor and immune heterogeneity and advancing biomarker discovery for precision oncology.

### 3.2 Spatial transcriptomics

Spatial transcriptomics offers a novel perspective by mapping gene expression within the spatial context of tissue architecture. This technology is particularly valuable for examining how the TME shapes tumor progression and response to therapy. Spatial mapping reveals distinct molecular niches within BM, such as areas enriched with chemokines like CXCL12 that recruit immune cells (65, 66). By integrating spatial data with transcriptomic profiles, researchers can identify localized immune evasion mechanisms and develop targeted therapies to overcome them (67, 68). Future applications may include combining spatial transcriptomics with advanced imaging modalities for a comprehensive understanding of tumor biology (69).

### 3.3 Proteomics and MS

Proteomics, driven by advancements in MS, provides detailed insights into the protein landscape of BM. MS-based approaches detect critical cytokine- and growth factor-related proteins, such as VEGF, MMPs, and ICAM-1, which are involved in angiogenesis, tumor invasion, and immune modulation (23, 59). Proteomics also uncovers post-translational modifications, such as phosphorylation of cytokine receptors, which can influence therapeutic responses (70, 71). By integrating proteomic findings with genomic and transcriptomic data, researchers can refine therapeutic strategies and identify new biomarkers for treatment personalization (72–74).

### 3.4 Liquid biopsy technologies

Liquid biopsy technologies provide non-invasive methods for tracking disease progression and treatment responses in real-time. These approaches analyze ctDNA, Exs, and cytokines to monitor tumor dynamics. For instance, circulating cytokines like IL-6, TNF-α, and VEGF serve as markers of systemic inflammation and immune activity, correlating with therapy resistance and tumor progression (75, 76), carrying cytokine-related proteins and RNA fragments reflect the molecular state of tumors and hold promise for biomarker discovery (77, 78). Despite challenges in assay sensitivity and specificity, advancements in liquid biopsy technologies are improving their reliability and potential for integration into routine clinical workflows (17, 79).

Emerging technologies, including NGS, scRNA-seq, spatial transcriptomics, proteomics, and liquid biopsies, are transforming biomarker discovery for BM (80). These innovations enhance our understanding of tumor biology and provide tools for developing personalized and adaptive immunotherapy strategies (81, 82). Future efforts should focus on validating these biomarkers in clinical settings and addressing challenges related to standardization, cost, and accessibility (83). Collaborative approaches combining technological innovation and interdisciplinary research are critical for advancing biomarker-driven precision oncology (84, 85).

## 4 Predictive indicators of immunotherapy response

Understanding and identifying predictive indicators of immunotherapy response are essential for optimizing treatment strategies for BM (52, 86). These indicators guide clinicians in selecting appropriate patients and help tailor treatment regimens to enhance efficacy. This section explores predictive indicators, including clinical, molecular, immunological, and imaging through the lens of cytokine and growth factor activity, while incorporating recent technological advances that enable more precise immune profiling.

### 4.1 Clinical predictors

Clinical predictors include demographic and treatment-related variables that influence immunotherapy outcomes. Factors such as age, performance status, and prior treatments are routinely evaluated in clinical settings. Elevated cytokines like IL-6 and TNF-α are correlated with systemic inflammation and poorer prognoses (87). Younger patients with a good Eastern Cooperative Oncology Group (ECOG) score (0–1) generally demonstrate better outcomes, likely due to preserved immune system (88). Additionally, prior therapies such as chemotherapy or targeted agents may modulate the immune landscape and thus affect responsiveness to ICIs (89). Recent evidence suggests that low-dose chemotherapy may act as a priming agent, enhancing immunogenicity and improving responsiveness to ICIs in patients with BM (90). Additional studies also indicate that low-dose chemotherapy may serve as an immunogenic primer, boosting antigen presentation and improving the efficacy of subsequent immunotherapy in BM patients (91). These insights underscore the importance of individualized treatment planning based on clinical predictors and their interplay with immune mechanisms.

Identifying these predictors enables effective patient stratification and personalized treatment plans to optimize therapeutic outcomes. The key predictors of immunotherapy response, categorized into clinical, molecular, immunological, and imaging domains, are summarized in **Table 2**, along with specific examples and their implications for treatment strategies.

**Table 2:** Predictive indicators of immunotherapy response in BM.

Predictor type	Specific examples	Implications for immunotherapy	References
Clinical predictors	Age and performance status (e.g., ECOG 0-1)	Younger patients and those with good ECOG status typically respond better due to a more robust immune system.	(92, 93)
	Prior treatments (e.g., chemotherapy, targeted therapy)	Prior treatment can alter the immune contexture and impact ICI responsiveness.	(89, 92)
	Low-dose chemotherapy priming	Enhances immunogenicity and may improve checkpoint blockade efficacy in BM	(90, 91)
Molecular predictors	VEGF, IL-6, and TGF-β pathway alterations	Associated with angiogenesis, immune evasion, and resistance to ICIs	(23, 48)
	Tumor Mutational Burden (TMB), MSI-H	Predict enhanced efficacy of PD-1/PD-L1 blockade in melanoma and lung cancer BM	(94, 95)
Immunological predictors	PD-L1 expression	High PD-L1 expression correlates with improved response to PD-1/PD-L1 inhibitors in NSCLC, melanoma BMs	(96, 97)
	TILs, CD8^+^ CTLs	Increased infiltration linked to better survival and ICI response	(98, 99)
Imaging predictors	PET tracers for IL-6 and TNF-α activity	Reflects inflammation; high signal may indicate immune resistance; low signal may reflect successful modulation	(100, 101)
	Radiomic: texture and volumetric metrics	Quantitative imaging markers associated with ICI response and intratumoral heterogeneity	(102, 103)

ECOG, Eastern Cooperative Oncology Group; ICI, immune checkpoint inhibitor; VEGF, vascular endothelial growth factor; TMB, tumor mutational burden; MSI-H, microsatellite instability-high; PD-L1, programmed death-ligand 1; TILs, tumor-infiltrating lymphocytes; CTLs, cytotoxic T lymphocytes; NSCLC, non-small cell lung cancer; BM, brain metastases.

### 4.2 Molecular predictors

Molecular predictors reveal intrinsic tumor properties that influence immunotherapy response. Key alterations in cytokine and growth factor pathways, such as VEGF, IL-6, and TGF-β drive, angiogenesis, immune escape, and resistance to ICIs (42, 48). Gene expression profiles, including EGFR, ALK, and BRAF mutations, are linked to differential responses to ICIs (104). Furthermore, tumors with high tumor mutational burden (TMB) or microsatellite instability-high (MSI-H) status indicate increased neoantigen loads, often translating into better responses to PD-1/PD-L1 inhibitors (105).

New technologies such as NGS enable deep genomic profiling of these alterations, while transcriptomic approaches allow for the identification of cytokine-driven gene expression programs. This facilitates personalized treatment strategies that target not only mutations but also aberrant signaling pathways relevant to immune modulation (13, 54, 57).

### 4.3 Immunological predictors

Immunological predictors provide insights into baseline immune competence and tumor-immune interactions. Biomarkers like PD-L1expression and tumor-infiltrating lymphocytes (TILs), particularly CD8^+^ cytotoxic T lymphocytes (CTLs), are well-established indicators of ICI response (33, 106, 107). Beyond surface marker expression, recent advances in single-cell RNA sequencing (scRNA-seq) and spatial transcriptomics allow for the high-resolution mapping of immune cell subtypes and their spatial distribution in the TME. These tools can uncover cytokine-rich niches or immunosuppressive zones, offering a nuanced understanding of immune heterogeneity in BMs. For example, IL-10 and IFN-γ signaling profiles can be inferred from scRNA-seq datasets, helping to predict ICI sensitivity at the single-cell level (108).

### 4.4 Imaging predictors

Imaging biomarkers offer a non-invasive tool to evaluate tumor burden, immune activity, and treatment response in BM. Functional imaging techniques, particularly positron emission tomography (PET) and magnetic resonance imaging (MRI), provide quantitative data on tumor metabolism, structural heterogeneity, and the immunologic landscape (109).

PET tracers such as ^18^F-fluorodeoxyglucose (^18^F-FDG) are commonly used to measure glucose metabolism in BM, correlating with tumor aggressiveness and immune evasion, particularly in NSCLC- and melanoma-derived BM. More advanced tracers like ^89^Zr-labeled atezolizumab, a radiolabeled anti-PD-L1 antibody, have been shown early promise in evaluating immune checkpoint activity and predicting ICI response (110, 111). Additionally, PET tracers targeting cytokine signaling, such as ^64^Cu-IL-6 and ^68^Ga-TNF-α analogs, visualize inflammatory niches and immune cell infiltration, serving as surrogate markers of immune activation or suppression (101, 112).

High uptake of these tracers suggests immune-active or cytokine-driven inflammatory regions, while reduced uptake may reflect effective immune modulation or tumor regression post-treatment (113, 114). On the structural imaging front, MRI-derived radiomic features such as texture, entropy, and volumetric indices are being explored for their predictive value in ICI responsiveness (115). These features may indicate underlying biological processes like necrosis, edema, or immune infiltration, aiding in patient stratification.

Integrating these imaging biomarkers into clinical practice enables real-time monitoring of treatment efficacy, facilitates adaptive therapy adjustments, and supports early identification of responders versus non-responders. Alongside clinical, molecular, immunological markers, and imaging predictors play a vital role in driving precision immunotherapy in BM (116, 117). Continued validation and integration into routine workflows are essential to fully realize the potential of personalized oncology in this complex setting (118).

## 5 Technological advances in biomarker discovery

The rapid progress in molecular profiling tools and high-throughput platforms has significantly enhanced our ability to discover and validate biomarkers for BM, particularly in the context of immunotherapy (7, 13). These innovations improve the precision and efficacy of cancer treatments by enabling the identification of cytokine- and growth factor-related biomarkers. This section focuses on the applications of NGS, single-cell technologies, MS, proteomics, and liquid biopsies in advancing biomarker discovery (25, 54).

### 5.1 NGS and single-cell technologies

NGS has transformed biomarker discovery by enabling comprehensive analysis of genetic alterations in cytokine and growth factor pathways, such as VEGF, IL-6, and TGF-β (13, 42). By providing detailed genomic profiles, NGS supports personalized treatment strategies, particularly for predicting immunotherapy responses and refining patient stratification (119).

ScRNA-seq complements NGS by offering insights into tumor heterogeneity and the immune microenvironment at a single-cell resolution. It identifies cytokine-producing cells, including TAMs secreting IL-10 or TGF-β and CD8^+^ T cells producing IFN-γ, which influence immunotherapy outcomes (11, 63, 120). The integration of bulk and single-cell data provides a comprehensive view of tumor biology, facilitating the discovery of biomarkers that address the complexities of BM and their resistance to therapy (36, 63).

### 5.2 Proteomics and MS

Proteomics, driven by MS, enables high-sensitivity analysis of protein expression, post-translational modifications, and protein-protein interactions. This approach has been pivotal in identifying biomarkers linked to tumor progression and therapeutic responses (121, 122). For instance, MS-based techniques have detected cytokine-induced proteins, such as MMPs and VEGF, that are critical for angiogenesis and immune modulation (23, 42, 123).

The ability of MS to detect post-translational modifications, like phosphorylation of cytokine receptors, provides deeper insights into protein functionality within the TME (124). Integrating proteomic data with genomic and transcriptomic profiles allows researchers to develop multidimensional biomarkers, enhancing the precision of personalized immunotherapy strategies (13).

### 5.3 Liquid biopsies

Liquid biopsies represent a non-invasive method for monitoring disease progression and treatment response. These technologies analyze ctDNA, exosomes, and cytokines present in bodily fluids, offering real-time insights into tumor dynamics (125). Liquid biopsies are particularly advantageous for their reduced invasiveness and ability to enable repeated sampling to track tumor evolution and therapeutic response.

Advanced assays quantify circulating cytokines and chemokines, such as IL-6, TNF-α, and CXCL12, which reflect the immune microenvironment and tumor progression (126, 127). Additionally, exosomes containing cytokine-related proteins and RNA fragments provide valuable insights into treatment resistance and immune activity (77, 128). Despite challenges in sensitivity and specificity, ongoing advancements in assay technologies improve the accuracy of liquid biopsies, making them indispensable tools for personalizing immunotherapy (38, 41).

**Figure 2:**
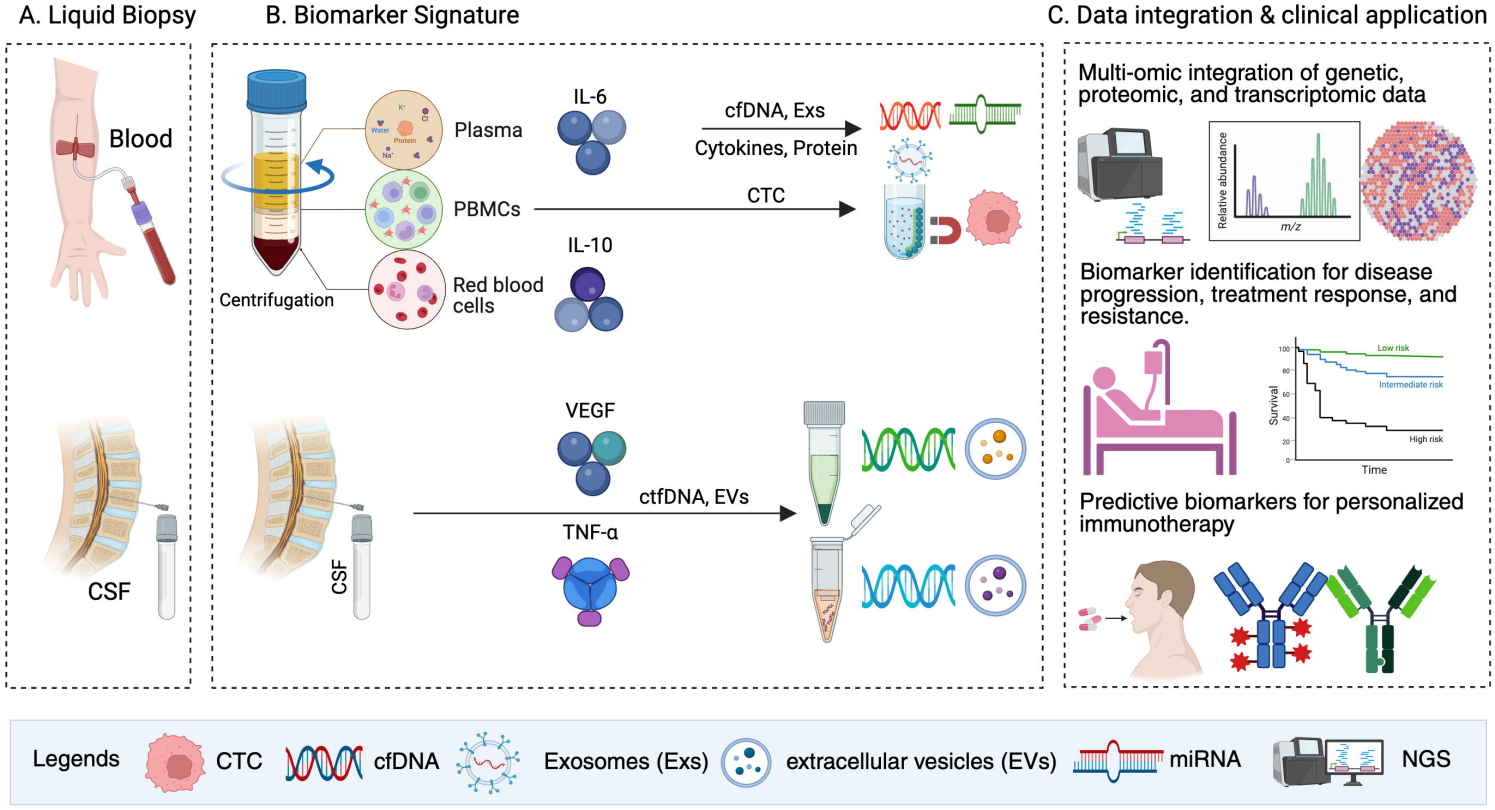
Liquid biopsy and biomarker discovery for personalized immunotherapy. This figure illustrates a streamlined workflow for liquid biopsy and cytokine/growth factor application. **(A)** Liquid biopsy: Blood and CSF are collected as sources of biomarkers. **(B)** Biomarker signature: Following centrifugation, plasma, PBMCs, and red blood cells are analyzed for key biomarkers such as IL-6, IL-10, VEGF, and TNF-α. Analytes including cfDNA, CTCs, Exs, and EVs are extracted for profiling. **(C)** Data integration and clinical application: multi-omic integration of genetic, proteomic, and transcriptomic data enables the identification of predictive biomarkers associated with disease progression, treatment response, and resistance. These insights inform treatment decisions and support personalized immunotherapy strategies.

**Figure 2** illustrates the workflow of liquid biopsy and proteomics-driven biomarker discovery, from sample collection and preparation to advanced molecular analyses and clinical applications, emphasizing its potential to revolutionize precision oncology.

Technological advancements, including NGS, scRNA-seq, MS, and liquid biopsies, drive significant progress in biomarker discovery for BM (129). By deepening our understanding of tumor biology and the immune microenvironment, these innovations pave the way for adaptive and personalized immunotherapy strategies (41, 130–132). Continued research and integration of these technologies into clinical workflows are critical for improving patient outcomes and advancing precision oncology (21, 133).

## 6 Challenges and future directions

The field of biomarker discovery for immunotherapy in BM is rapidly evolving. However, numerous challenges related to cytokine and growth factor biomarkers remain. Addressing these challenges across technical, biological, and clinical domains is essential. Furthermore, future research must prioritize integrating these biomarkers into clinical practice to achieve meaningful advancements.

### 6.1 Technical challenges

Quantifying low-abundance cytokines and growth factors in complex biological matrices like blood or cerebrospinal fluid presents significant technical challenges (42, 134). These molecules exist at picomolar concentrations, necessitating the use of highly sensitive and specific detection methods, such as advanced multiplex assays or MS-based techniques (130, 135). Additionally, the structural similarity among many cytokines and growth factors complicates assay specificity, requiring the development of next-generation detection platforms (136). Standardizing protocols across laboratories is crucial to improve reproducibility and facilitate biomarker validation (137). Automated workflows and high-throughput technologies should be prioritized to address scalability and minimize variability in biomarker quantification, thereby expediting their clinical adoption.

### 6.2 Biological challenges

The variability in cytokine and growth factor expression, driven by tumor heterogeneity and systemic inflammation, complicates their utility as reliable biomarkers. BM exhibit significant spatial and temporal heterogeneity, resulting in distinct cytokine profiles that evolve in response to treatment interventions and tumor-immune dynamics (84). For instance, IL-6 overexpression may signify both tumor progression and systemic inflammation, complicating its interpretation as a biomarker (13, 138). Additionally, the dynamic nature of cytokines and growth factors necessitates longitudinal studies to account for fluctuations during disease progression or therapy response. Technologies like scRNA-seq and spatial transcriptomics are essential for providing high-resolution insights into cytokine-producing cells and their spatial distribution, revealing actionable targets for therapeutic interventions (139).

### 6.3 Clinical challenges

Overcoming logistical, ethical, and regulatory barriers is critical for integrating cytokine and growth factor biomarkers into clinical practice. Biomarker testing workflows must be streamlined to ensure cost-effectiveness and avoid delays in patient care (140). Ethical considerations, such as obtaining informed consent and addressing disparities in access to advanced diagnostic technologies, must be addressed to promote equitable healthcare delivery (141). From a regulatory perspective, cytokine and growth factor assays must meet stringent criteria for analytical validity, clinical utility, and scalability (54). Collaborative efforts among researchers, clinicians, and regulatory agencies are necessary to demonstrate the clinical relevance of these biomarkers in predicting treatment responses and guiding therapy (142). Such collaborations will facilitate broader adoption and regulatory approval.

**Figure 3:**
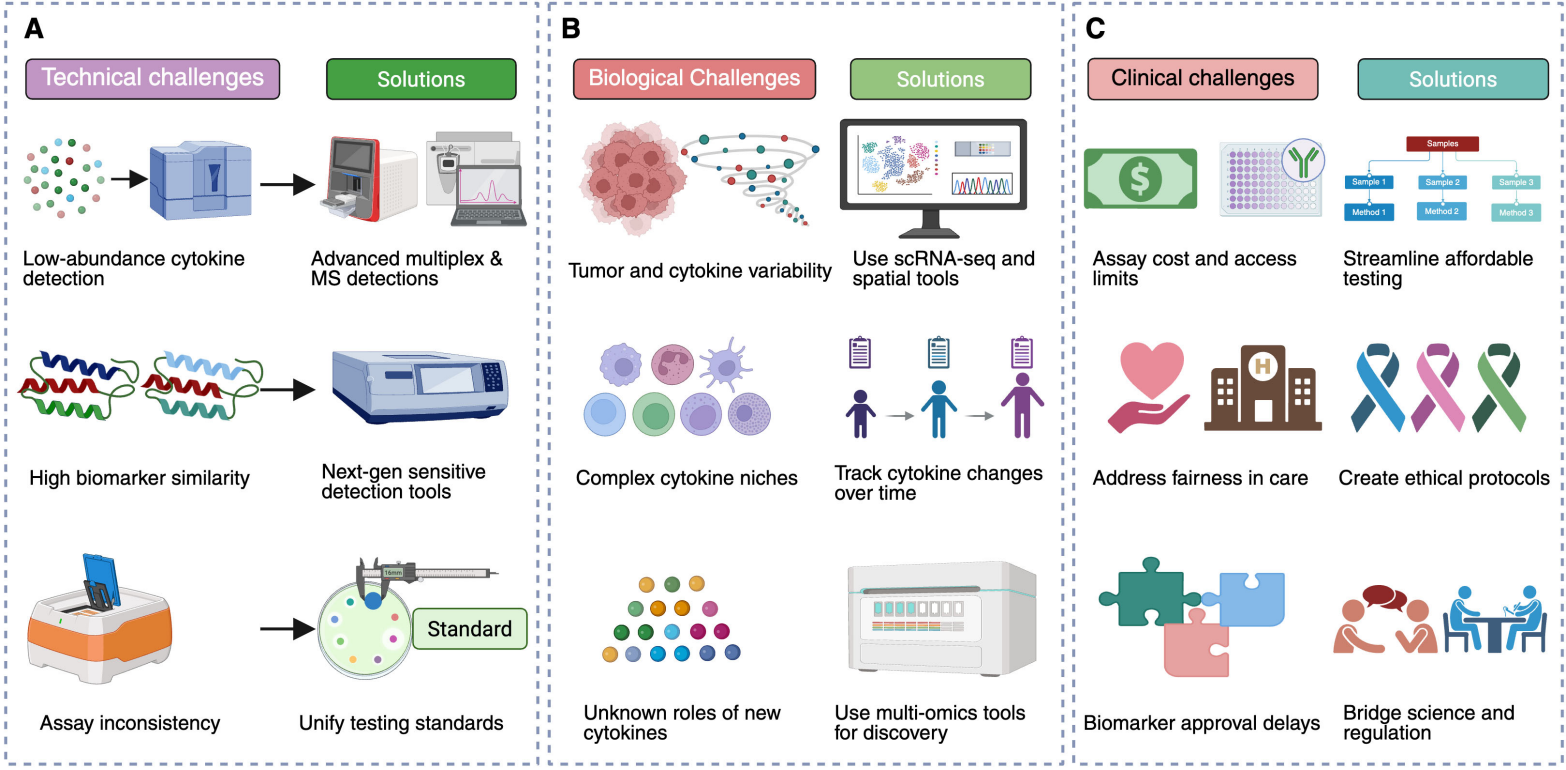
Challenges and solutions in biomarker discovery for immunotherapy. This figure summarizes key technical, biological, and clinical challenges in biomarker discovery for immunotherapy and presents potential solutions to address them. **(A)** Technical challenges include the detection of low-abundance cytokines and growth factors, high structural similarity among biomarkers, and lack of assay standardization. Solutions involve utilizing advanced multiplex and MS-based assays, developing next-generation detection platforms with improved sensitivity, and standardizing protocols across laboratories. **(B)** Biological challenges stem from tumor heterogeneity, fluctuating cytokine levels, complexity within cytokine-driven immune niches, and limited knowledge of novel cytokines. Proposed solutions include employing scRNA-seq and spatial transcriptomics, conducting longitudinal studies to track cytokine dynamics, and exploring multi-omic assays for novel discoveries. **(C)** Clinical challenges include logistical barriers such as assay cost and accessibility, ethical concerns in equitable healthcare delivery, and regulatory hurdles for biomarker validation. Solutions emphasize prioritizing scalable, cost-effective workflows, building ethical frameworks for advanced testing, and fostering collaboration among researchers and regulatory bodies to streamline biomarker validation.

The integration of technological, biological, and clinical strategies is essential for overcoming these challenges and advancing biomarker-driven immunotherapy. **Figure 3** provides an overview of key challenges in biomarker discovery and corresponding solutions aimed at improving the clinical utility of cytokine and growth factor biomarkers.

### 6.4 Future research directions

Future research should focus on discovering novel cytokine and growth factor biomarkers, particularly underexplored molecules like IL-10, TGF-β, and CXCL12, which influence immune evasion and tumor progression (143). Leveraging multi-omic approaches, including genomics, transcriptomics, proteomics, and metabolomics, can elucidate cytokine-driven pathways and their implications for immunotherapy (144). Rigorous clinical validation through prospective trials and real-world studies is critical for establishing the, and prognostic value of these biomarkers (145). Artificial computational tools, such as AI and ML, should be employed to analyze complex multi-omic datasets, uncovering patterns that might otherwise remain undetected (146). Moreover, developing scalable and user-friendly assays is vital to ensure these biomarkers can be adopted across diverse healthcare settings.

Despite substantial progress in cytokine and growth factor biomarker discovery, addressing technical, biological, and clinical challenges is essential to fully realize their potential (13). By prioritizing the development of robust detection platforms, understanding cytokine variability, and integrating validated biomarkers into clinical workflows, the field can drive innovation in personalized immunotherapy strategies (13, 54, 147). These efforts promise not only improved outcomes for patients with BM but also significant advancements in precision oncology.

## 7 Conclusion

The evolving landscape of immunotherapy biomarkers in BM underscores the central role of cytokines and growth factors in modulating immune responses within the TME (52, 148). Through this review, we synthesized evidence across genomic, proteomic, immunological, circulating, and microenvironmental domains, highlighting how these biomarkers inform prognosis and therapeutic decision-making (13, 54).

### 7.1 Key findings

Cytokine and growth factor pathways influence tumor progression, immune evasion, and treatment response (42). Advances in high-throughput technologies such as NGS, scRNA-seq, spatial transcriptomics, and liquid biopsies have enabled deeper profiling of immune landscapes, facilitating biomarker discovery and risk stratification (149).

### 7.2 Translational potential

To translate these findings into clinical impact, future efforts must address key barriers, including assay variability, tumor heterogeneity, and limited validation across populations (13, 54). Integrating multi-omic data will enhance biomarker precision and support personalized immunotherapy approaches (144, 150, 151).

### 7.3 Future directions

Moving forward, interdisciplinary collaboration is essential to optimize biomarker platforms, ensure regulatory alignment, and drive multicenter validation (84, 152, 153). The integration of AI and machine learning will further refine predictive models, accelerating the clinical adoption of biomarker-driven strategies for BM (154–156).
